# Effects of Alpha Lipoic Acid Supplementation on Serum Levels of Oxidative Stress, Inflammatory Markers and Clinical Prognosis among Acute Ischemic Stroke Patients: A Randomized, Double Blind, TNS Trial

**DOI:** 10.34172/apb.2020.034

**Published:** 2020-02-18

**Authors:** Sheyda Shaafi, Soraiya Ebrahimpour-Koujan, Mohammad Khalili, Seyad Morteza Shamshirgaran, Mazyar Hashemilar, Aliakbar Taheraghdam, Seyed Kazem Shakouri, Elyar Sadeghi Hokmabadi, Yaeghoub Ahmadi, Mehdi Farhoudi, Nasim Rezaeimanesh, Daryoush Savadi Osgouei

**Affiliations:** ^1^Department of Neurology, School of Medicine, Tabriz University of Medical Sciences, Tabriz, Iran.; ^2^Neurosciences Research Center, Tabriz University of Medical Sciences, Tabriz, Iran.; ^3^Students Scientific Research Center, Tehran University of Medical Sciences, Tehran, Iran.; ^4^Multiple Sclerosis Research Center, Neuroscience institute, Tehran University of Medical Sciences, Tehran, Iran.; ^5^Healthy Aging Research Center, Neyshabur University of Medical Sciences, Neyshabur, Iran.; ^6^Physical Medicine and Rehabilitation Research Center, Tabriz University of Medical Sciences, Tabriz, Iran.

**Keywords:** Alpha lipoic acid, Inflammation, Oxidative stress, Stroke

## Abstract

***Purpose:*** Stroke is one of the most common conditions causing death. There have been few studies examining the effects of alpha lipoic acid (ALA) on stroke patients. In this regard, the present randomized controlled clinical trial was conducted to examine the effects of ALA supplementation on serum albumin, and inflammatory and oxidative stress markers in stroke patients.

***Methods:*** The present paralleled randomized controlled clinical trial involved 42 stroke patients who were over 40 years and under enteral feeding. The participants were randomly assigned into two groups and finally 40 patients completed the study. Patients in alpha lipoic acid group (n=19) took 1200 mg ALA supplement daily along with their meal, and participants in control group (n=21) underwent the routine hospital diet for 3 weeks. Fasting blood samples were obtained and albumin, oxidative stress, and inflammatory indices were assessed at baseline, as well as at the end of the trial.

***Results:*** After 3 weeks, treatment of patients with ALA led to a significant decrease in tumor necrosis factor alpha (TNF-α) and interleukin 6 (IL-6) levels (*P*=0.01) compared to baseline. But serum levels of albumin, total antioxidant capacity (TAC), malondialdehyde (MDA), highsensitivity C-reactive protein (hs-CRP), IL-6 and TNF-α did not change significantly vs. control group (*P*>0.05).

***Conclusion:*** ALA did not significantly change the serum levels of albumin and inflammatory as well as antioxidant capacity indices in stroke patients compared with the control group. More clinical trials with large sample sizes and long duration are needed to clarify the effects of ALA on these patients.

## Introduction

Stroke is the second leading cause of death across the world.^1^ It has various clinical, social, and economic burdens and its management demands a comprehensive effort by both basic scientists and clinicians.^2^ Ischemic stroke which is caused by either a sudden reduction in brain blood flow or complete blockage of it, accounts for 85% of all strokes.^3^

Despite sophisticated medical management and neurosurgical techniques, the mortality rate of stroke is remarkable.^4^ Wide lines of studies have focused on well-defined risk factors of stroke and its management strategies. Both life style and diet-related factors contribute to its high rate mortality.^1^ Better understanding of risk factors would improve treatment outcomes and prognosis of patients. It has been strongly clarified that oxidative stress is a fundamental mechanism causing brain injury in stroke patients.^5^ In fact, increasing levels of reactive oxygen species (due to lower levels of antioxidant defense and higher levels of oxygen after reperfusion), oxidizing lipids, and iron induce oxidative stress. Therefore, the increase in ROS production and consequently in oxidative stress, as well as the increase in inflammatory mediators are the earliest and the most potent mechanisms involved in brain damage during the stroke.^1,5^ Modulation of free radicals derived from metabolic processes is one of the most important strategies in preventing reperfusion injury during stroke. This process is mainly mediated by antioxidants of the body.^6^ Alpha lipoic acid (ALA, 1,2-dithiolane-3-pentanoic acid), a neuro-protective and neuro-restorative antioxidant, with the ability to cross through blood–brain barrier, is a strong free radical scavenger in the brain.^7,8^ There are recent interests to assess the effects of ALA in central nervous system diseases such as Parkinson’s disease, Multiple sclerosis, Alzheimer’s disease, and spinal cord injury.^9-11^ Lipoic acid protects the brain against reperfusion injury in the early stages of cerebral ischemia.^12^ Primary experimental evidence has revealed that treatment of rats in cerebral artery occlusion condition with lipoic acid protects the brain against ischemic stroke by modulating several inflammatory pathways.^3,4,12-14^ Despite the positive results from a clinical trial on protective effects of lipoic acid on clinical outcomes after acute ischemic stroke,^6^ there is no evidence confirming its role in modulating the inflammatory process and oxidative stress in stroke patients. It seems that some clinical trials with basic trends are required to clarify the effects of lipoic acid on the modulation of the levels of free radicals and oxidative stress as main mediators in stroke. It is especially important in Iran where the prevalence of stroke is rising and this severely affects the patients’ quality of life which is effective in controlling the complications of the disease. Therefore, this randomized controlled clinical trial was conducted to examine the effects of ALA supplementation on serum albumin, and inflammatory and oxidative stress markers in stroke patients.

## Material and Methods

### Participants

**Figure 1:**
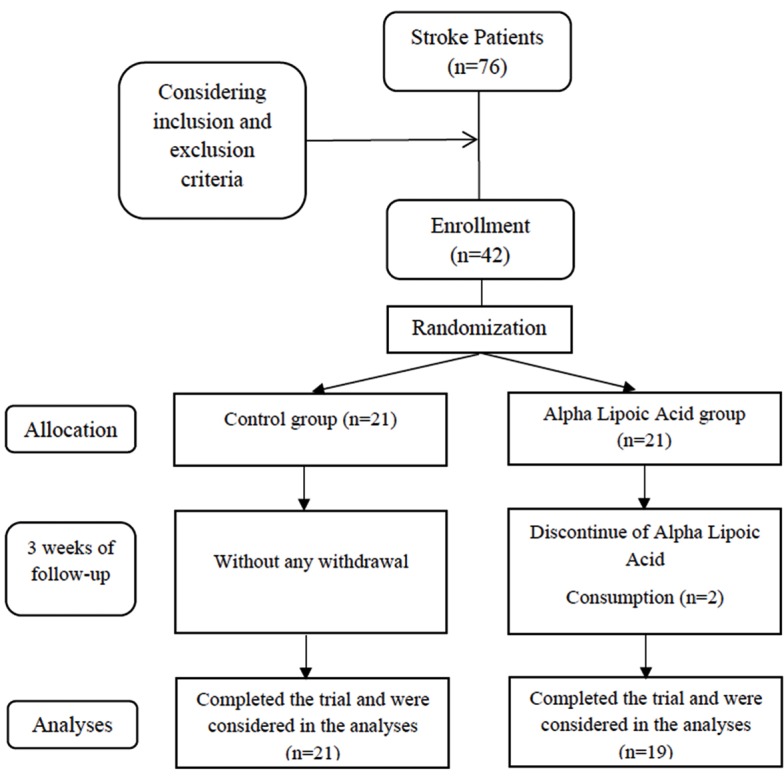
Study participants’ diagram.

The target population of the present randomized clinical trial (RCT) was patients with acute ischemic stroke of cerebral hemisphere who had referred to Imam Reza hospital, Tabriz University of Medical Sciences, Tabriz, Iran (Tabriz stroke registry project). The patients were screened according to the inclusion and exclusion criteria and 42 individuals of them were involved to this study, among which 40 patients completed the study (Figure 1). The patients who met the following criteria were eligible to include in the study: those who were admitted for the first time or diagnosed with acute ischemic stroke, those over 40 years, those whose National Institutes of Health Stroke Scale (NIHSS) scores were less than 20, and those for whom the enteral or oral feeding was done in first 48 hours of admission. The exclusion criteria included, being in vegetative state, having chronic conditions (including kidney failure, undergoing dialysis, liver failure and cirrhosis in the admission, seizure, cancer, gastrointestinal bleeding, uncontrolled diabetes, having any history of heart attack during past three month, respiratory disease, heart failure, and hematologic disease), any changes in diagnosis and feeding methods, taking enteral feeding less than 10 days, taking immune-suppressive medications, abusing alcohol and drugs, death in the first 10 days of admission, and lactose intolerance. Patients were assigned to intervention and control groups by block randomization method. Randomization was done by computer-generated random numbers. Allocation of participants into the groups was done by a third person who was not directly involved in the project. Intervention group took 1200 mg ALA supplement daily along with the ordinary meal and participants in control group underwent the routine hospital diet for 3 weeks. All participants received kitchen made feeding with composition of 1.5 g/day protein and 25 kcal/kg energy per day. The ALA supplement was produced by Caren co. We did all measurements both at baseline and at the end of the trial. We calculated the required sample size based on data from a previous study^15^ by considering serum interleukin 6 (IL-6) as a key dependent variable, type I error of 0.05, and study power of 80%. Based on suggested formula for clinical trials and taking into account a possible dropout of 20%, we came up with the sample size of 20 stroke patients for each group.

### Study design

This project was done in accordance with the guidelines laid down in the Declaration of Helsinki (1964). Individual questionnaires including demographic characteristics, past medical and drug history, as well as socio-economic status were completed for each patient by their family through comprehensive face-to-face interviews.

### Biochemical measurements

Prior to the intervention and at the end of the trial, after at least 8 hours overnight fasting, 7 mL of venous blood samples were obtained from each patient. Blood samples were immediately centrifuged at 4000 rpm for 15 minutes in aseptic condition. Serum samples were separated in 1 mL micro-tubes and were immediately stored at -80°C. Serum levels of albumin, total antioxidant capacity (TAC), malondialdehyde (MDA), IL-6, high-sensitivity C-reactive protein (hs-CRP), and tumor necrosis factor alpha (TNF-α) were measured using enzyme-linked immunosorbent assay (ELISA) kits at the baseline and endpoint of the trial.

### Clinical prognosis measurements

NIHSS is a systematic method that provides a quantitative measure of stroke-induced neurological damage and is generally designed for patients with acute stroke. The scale is comprised of 15 items and covers the results of stroke patients’ neurological examination and assesses the effect of acute stroke on the level of consciousness, language, eye movements, motor force, ataxia, and sensorium. Each item is scored either on a 3 or 5 point scale, where zero represents the normal state and upper limit is 42. Also, modified Rankin Scale (mRS) is comprised of 6 levels and evaluates independence rather than the performance of specific tasks. The scale consists of 6 grades, ranging from 0 to 6, where zero is associated with no symptoms, five represents severe disability, and 6 means death.

### Statistical analyses

Statistical analyses of all data were performed using SPSS software version 21. Data results were expressed as mean ± standard deviation (±SD). We used the one-sample Kolmogorov-Smirnov test for assessing the normality of the data distribution. Baseline characteristics of the patients in the alpha lipoic acid and control groups were compared using independent sample *t* test and chi-square test for quantitative and qualitative variables, respectively. Paired *t* test analysis (following the intention to treat principle) was used to identify any differences between the two groups after intervention. The *P* value<0.05 was considered statistically significant.

## Results

All 40 patients (19 patients in alpha lipoic acid group and 21 patients in control group) completed the 3-week trial. No patient reported adverse effects or symptoms with ALA supplementation during the trial. In lipoic acid group, 68.4 % of the participants were men and 31.6 % were women (13 males and 6 females). In control group 81.0 % of the participants were men and 19.0 % were women (17 males and 4 females). The demographic characteristics and anthropometric measures of the participants are shown in Table 1.There were no significant differences between the two groups regarding baseline variables including age, sex, marital status, education, smoking, co-morbidities, and lesion location at the beginning of the study (*P* ˃ 0.05).

**Table 1:** General and clinical characteristics of study participants

	**Groups**		
	**Alpha lipoic acid (n=19)**	**Control** **(n=21)**	***P*** ^a^
Age (y)	71.8±10.8	71.9±5.9	0.97
Females (%)	31.6	19.0	0.36
Married (%)	94.7	100	0.29
Educated (%)	26.3	42.9	0.27
Having co-morbidity (%)	84.2	81	0.71
Smoking (%)	26.3	38.1	0.19
Lesion area	-	-	0.11
Right hemisphere (%)	42.1	42.9	-
Left hemisphere (%)	47.4	38.1	-
Brainstem (%)	10.5	19.0	-

MET, metabolic equivalents. All values are Mean±SD unless indicates. ^a^ *P* values were obtained from independent student’s t test or χ2 test, where appropriate.

The serum levels of albumin, TAC, MDA, TNF-α and hs-CRP before and after the intervention and their variations are shown in Table 2. There were just significant differences in term of MDA between two groups at the baseline (*P* =  0.01) and endpoint (*P* =  0.03) of study. Supplementation with lipoic acid resulted in no significant changes on antioxidant capacity compared with control group. Also, in term of other inflammatory factors the changes after 3 weeks were not significantly different between study groups (*P* ˃ 0.05).

**Table 2:** Serum oxidative stress and inflammatory parameters of stroke patients at the baseline and after 3 weeks intervention with alpha lipoic acid

**Variable**	**Measurement period**	**Alpha lipoic acid group (n=19)**	**Control group (n=21)**	***P*** **value for between groups**
Albumin (mg/dL)	Baseline	2.94±0.78	3.32±0.79	0.13^a^
	After intervention	2.92±0.62	3.42±1.07	0.08^a^
	Change, MD (95%CI), *P* value ^b^	-0.01 (-0.14,0.11), 0.79	0.10 (-0.15,0.36), 0.41	0.63 ^a^
TAC (mmol/mL)	Baseline	3638.31±860.30	3835.14±881.83	0.48^a^
	After intervention	3837.95±888.13	3842.19±938.21	0.99^a^
	Change, MD (95% CI), *P* value^b^	199.63 (24.27,423.54), 0.07	7.05 (-125.33,139.42), 0.91	0.11^a^
MDA (nmol/mL)	Baseline	2.02±0.14	1.82±0.32	0.01^a^
	After intervention	2.05±0.26	1.83±0.38	0.03^a^
	Change, MD (95% CI), *P* value^b^	0.03 (-0.08,0.15), 0.57	0.01 (-0.12,0.14), 0.88	0.91^a^
hs-CRP (mg/dL)	Baseline	3.32±1.81	3.58±1.56	0.63^a^
	After intervention	3.16±1.51	3.47±1.43	0.51^a^
	Change, MD (95% CI), *P* value^b^	-0.16 (-0.37,0.4), 0.11	-0.11 (-0.25,0.03), 0.11	0.92^a^
TNF-α (pg/mL)	Baseline	20.50±13.91	18.09±13.14	0.57 ^a^
	After intervention	16.49±9.63	15.95±9.03	0.85^a^
	Change, MD (95% CI), *P* value ^b^	-4.00 (-7.10-0.91), 0.01	-2.14 (-5.51,1.23), 0.20	0.21^a^
IL-6 (pg/mL)	Baseline	8.39±6.05	7.31±5.81	0.57^a^
	After intervention	3.85±4.62	3.78±4.44	0.98^a^
	Change, MD (95% CI), *P* value^b^	-4.54 (-4.74,2.53), 0.01	-3.43 (-7.46,1.62), 0.01	0.54^a^

TAC: total antioxidant capacity; MDA: malondialdehyde; hs-CRP: high sensitivity C-reactive protein; TNF-α: tumor necrosis factor-α; IL-6: interleukin 6. The results are described as mean ± standard deviation (SD). ^a^ MD (95% CI), *P* value is reported based on the analysis of independent sample *t* test. ^b^ MD (95% CI), *P* value is reported based on the analysis of paired sample *t* test.

After 3 weeks, treatment of patients with ALA in intervention group, led to a significant decrease in TNF-α and IL-6 (*P* =  0.01) and not significant increase in TAC (*P* =  0.07) serum concentrations. There were no significant differences between baseline levels of albumin, MDA, and hs-CRP, and end point concentrations in alpha lipoic acid group (*P* >  0.05).

Table 3 provides the mean changes in clinical measurements of patients from the baseline to the end point. During study, there was a significant decrease in NIHSS and mRS in lipoic acid (7.10 ± 4.34 vs. 5.52 ± 3.58, *P* =  0.01), (1.73 ± 1.09 vs. 1.47 ± 0.96, *P* =  0.02) and control groups (5.28 ± 4.57 vs. 3.42 ± 1.07, *P* =  0.02), (1.85±1.49 vs. 1.47 ± 1.28, *P* =  0.04), respectively. However, there were no changes regarding the clinical prognosis indices between two groups.

**Table 3:** Clinical prognosis indexes of stroke patients at the baseline and after 3 weeks intervention with alpha lipoic acid

**Variable**	**Measurement period**	**Alpha lipoic acid group (n = 19)**	**Control group (n = 21)**	***P*** **value for between groups**
NIHSS	Baseline	7.10±4.34	5.28±4.57	0.21^a^
	After intervention	5.52±3.58	3.81±3.34	0.14^a^
	Change; MD (95% CI), *P* value ^b^	-1.57 (-0.74-2.28), 0.01	-1.47 (-0.15-0.36), 0.01	0.87 ^a^
mRS	Baseline	1.73±1.09	1.85±1.49	0.77^a^
	After intervention	1.47±0.96	1.47±1.28	0.99^a^
	Change; MD (95% CI), *P* value ^b^	-0.26 (-0.04-0.48), 0.02	-0.38 (-0.01-0.74), 0.04	0.57^a^

NIHSS: National Institutes of Health Stroke Scale; mRS: modified Rankin Scale. The results are described as mean ± standard deviation (SD). ^a^ MD (95% CI), *P* value is reported based on the analysis of independent sample *t* test. ^b^ MD (95% CI), *P* value is reported based on the analysis of paired sample *t* test.

## Discussion

Our study demonstrated that ALA supplementation for 3 weeks led to a significant decrease in serum TNF-α and IL-6 in intervention group, but no significant differences were founded on inflammatory and antioxidant biomarkers variation between intervention and control groups. To the best of our knowledge, this is the first clinical trial on the effects of ALA supplementation on inflammatory and oxidative stress biomarkers in stroke patients.

Neuro-protective effects of ALA had been studied frequently in central nervous system diseases.^10^ It is demonstrated that reactive oxygen scavenging capacity and ability to regenerate endogenous antioxidants necessary for repair systems relate to neuro-protective and neuro-restorative roles of lipoic acid, respectively.^10^ Our results, as the first study which examined the effects of ALA supplementation on anti-oxidant profile in stroke patients, are inconsistent with previous studies conducted on related issues. We did not observe significant effects of ALA supplementation on serum concentrations of TAC and MDA. It has been reported that TAC levels were improved in multiple sclerosis patients through ALA supplementation. However, in line with our results, they did not observe significant changes in MDA levels.^16^ It seems that the ischemic reperfusion in stroke patients leads to over production of ROS and consequently increase in the levels of oxidative stress mediators, several months after stroke.^17,18^ Therefore, short term supplementation with ALA may not be able to affect body anti-oxidant profile. It is recommended that all treatment procedures should be followed at least for 3 months.

Three-week supplementation with ALA led to a significant decrease in serum concentration of TNF-α and IL-6 in intervention group. However, there were no significant differences between two groups in terms of TNF-α, IL-6, and hs-CRP as well as serum levels of albumin (as a negative acute phase protein). Our results are not consistent with the findings of the previous trial on supplementation with ALA which showed a significant decrease in serum inflammatory cytokines in MS patients.^19^ The serum inflammatory markers usually remain high over months after stroke.^20,21^ Due to acute inflammation, the levels of negative phase proteins including albumin are also low in stroke patients over a month. Hypoalbuminemia is frequently reported among stroke patients and correlates with the pro-inflammatory pattern of serum protein electrophoresis.^22,23^ It seems that examining the changes in serum inflammatory indicators needs relatively long-term trials. It should also be mentioned that stroke patients suffer from various co-morbidities including hypertension, diabetes, etc, which are basically accompanied with chronic inflammation and increased levels of pro-inflammatory cytokines that interrupt their treatment. Therefore, comprehensive therapeutic strategies with long-term interventions should be developed.

There are numerous studies on stroke models that have examined its potential action mechanisms.^24^ As ROSs cause tissue injury leading to ischemic reperfusion in stroke, ROS scavengers are considered in CNS-protecting therapies.^25^ It has been shown that protective effects of ALA against cell death following stroke are mediated by increasing the antioxidant enzyme levels and scavenging ROS.^12,13^ Immediate injection of ALA decreases the infarct size in the rat partially via insulin receptor activation.^4,26^ Experimental evidence suggested that inhibition of peripheral TNF-α and down-regulation of brain microglial activation by ALA protect rat brain against ischemic stroke.^14^ It seems that expressions of nuclear factor erythroid 2-related factor 2 and heme oxygenase-1 are induced by ALA treatment that consequently decreases the intracellular ROS, infarct volume, brain edema, and oxidative damage, and promotes neurologic recovery in rats.^3^ ALA had a significant positive effect on NIHSS and mRS values but no statistically significant differences were found at baseline and after potential cofounder adjustment between two groups.

No side effects were reported for ALA supplementation in the present study. There are some limitations that should be considered in interpretation of our results. Although all patients were on the same hospital diet, individual intakes of patients were not considered. Therefore, the dietary intakes and nutritional status of patients were not evaluated which are important confounders affecting the inflammatory and oxidative stress indicators. Moreover, it seems that duration of supplementation was not long enough to examine the changes reported here. The small sample size was other factor affecting the findings. Thereafter, more clinical trials with large sample sizes in this regard are required.

## Conclusion

In conclusion, 3 weeks of supplementation with ALA did not have any significant effects on serum albumin, and oxidative stress and inflammatory biomarkers compared to control group in stroke patients. However, more clinical trials with large sample sizes and long duration are required to clarify the effects of ALA on stroke patients.

## Ethical Issues

The main goals of study were described for the first grade relatives of patients and written consent was obtained. The study was registered on the Iranian Registry of Clinical Trials website (identifier: IRCT2016061428450N1; http://www.irct.ir). The trial was approved by the bioethics committee of the Tabriz University of Medical Sciences (TBZMED.REC.1395.55).

## Conflict of Interest

Authors declare no conflict of interest in this study.

## Funding

This study received its financial support from Vice-Chancellor for Research of Tabriz University of Medical Sciences, Tabriz, Iran.
